# Diethyl 2-{(dibenzyl­amino)[4-(trifluoro­meth­yl)phen­yl]meth­yl}malonate

**DOI:** 10.1107/S1600536810010512

**Published:** 2010-03-27

**Authors:** Ihssan Meskini, Maria Daoudi, Jean-Claude Daran, Hafid Zouihri, Abdelali Kerbal

**Affiliations:** a Laboratoire de Chimie Organique, Faculté des Sciences Dhar el Mahraz, Université Sidi Mohammed Ben Abdellah, Fès, Morocco; b Laboratoire de Chimie de Coordination, 205 Route de Narbonne, 31077 Toulouse Cedex, France; c Centre National pour la Recherche Scientifique et Technique, Division UATRS, Rabat, Morocco

## Abstract

The asymmetric unit of the title compound, C_29_H_30_F_3_NO_4_, contains two independent mol­ecules. In each independent mol­ecule, one of two terminal ethyl groups is disordered over two conformations: the occupancies of major components were fixed at 0.53 and 0.64 in the two mol­ecules. In the crystal structure, weak inter­molecular C—H⋯O hydrogen bonds link mol­ecules into chains propagating along [10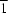].

## Related literature

For related compounds exhibitinging biological activity, see: Dayam *et al.* (2007 ▶); Patil *et al.* (2007 ▶); Ramkumar *et al.* (2008 ▶); Sechi, Carta *et al.* (2009 ▶); Sechi, Rizzi *et al.* (2009 ▶); Zeng, Zhang *et al.* (2008 ▶); Zeng, Jiang *et al.* (2008 ▶). For details of the synthesis, see: Pommier & Neamati (2006 ▶).
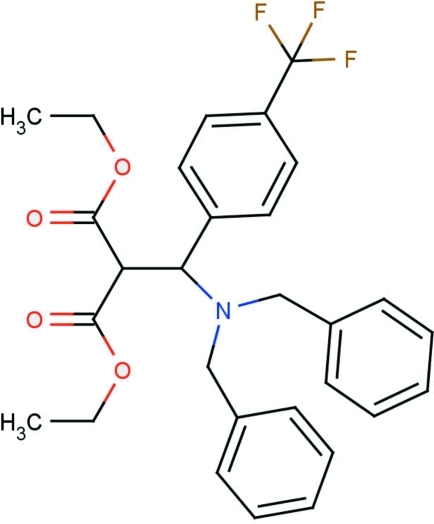


## Experimental

### 

#### Crystal data

C_29_H_30_F_3_NO_4_
*M* *_r_* = 513.54
Monoclinic, 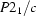

*a* = 13.4131 (3) Å
*b* = 23.6608 (5) Å
*c* = 17.3769 (3) Å
β = 96.826 (1)°
*V* = 5475.72 (19) Å^3^
*Z* = 8
Mo *K*α radiation
μ = 0.10 mm^−1^
*T* = 296 K
0.43 × 0.25 × 0.17 mm

#### Data collection

Bruker APEXII CCD detector diffractometer
74220 measured reflections
10790 independent reflections
6912 reflections with *I* > 2σ(*I*)
*R*_int_ = 0.041

#### Refinement

*R*[*F* ^2^ > 2σ(*F* ^2^)] = 0.060
*wR*(*F* ^2^) = 0.184
*S* = 1.02
10790 reflections
709 parameters
10 restraints
H-atom parameters constrained
Δρ_max_ = 0.51 e Å^−3^
Δρ_min_ = −0.41 e Å^−3^

### 

Data collection: *APEX2* (Bruker, 2007 ▶); cell refinement: *SAINT* (Bruker, 2007 ▶); data reduction: *SAINT*; program(s) used to solve structure: *SIR97* (Altomare *et al.*, 1999 ▶); program(s) used to refine structure: *SHELXL97* (Sheldrick, 2008 ▶); molecular graphics: *PLATON* (Spek, 2009 ▶); software used to prepare material for publication: *publCIF* (Westrip, 2010 ▶).

## Supplementary Material

Crystal structure: contains datablocks I, global. DOI: 10.1107/S1600536810010512/cv2702sup1.cif

Structure factors: contains datablocks I. DOI: 10.1107/S1600536810010512/cv2702Isup2.hkl

Additional supplementary materials:  crystallographic information; 3D view; checkCIF report

## Figures and Tables

**Table 1:** Hydrogen-bond geometry (Å, °)

*D*—H⋯*A*	*D*—H	H⋯*A*	*D*⋯*A*	*D*—H⋯*A*
C226—H226⋯O22^i^	0.93	2.60	3.323 (4)	135
C235—H235⋯O14^ii^	0.93	2.51	3.294 (4)	142

Symmetry codes: (i) 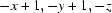; (ii) 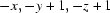.

**Fig. 1.**
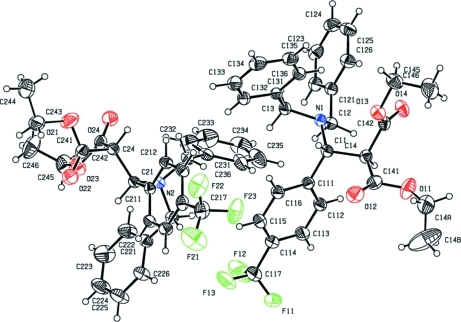
Two independent molecules of the title compound showing the atom-labelling scheme and 30% probability displacement ellipsoids. Only major parts of disordered ethyl groups are shown.

**Fig. 2.**
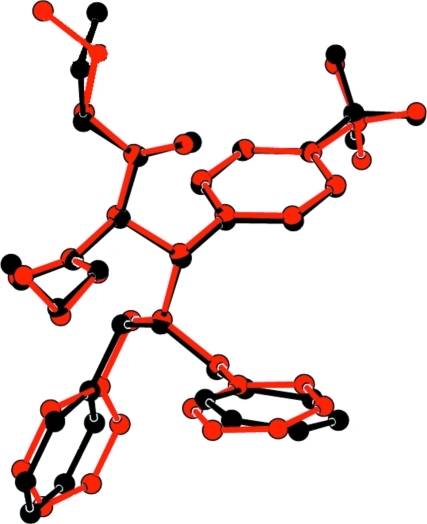
View showing the fitting of two independent molecules. Only major parts of disordered ethyl groups are shown.

## supplementary crystallographic information

### Comment

The rational design of new HIV-1 Integrase (H—I) inhibitors, validated target for chemotherapeutic intervention (Dayam *et al., *2007), is fundamentally based on intermolecular coordination between H—I / chemical inhibitor / metals (Mg^+2^ and Mn^+2^, co-factors of the enzyme) leading to formation of bimetallic complexes (Zeng, Zhang *et al.*, 2008; Sechi, Carta *et al.*, 2009). Thereby, several bimetallic complexes, in many cases exploring the well-known polydentate ligands, appear in this scenario as the most promising concept to employ in either enzyme / drug interaction or electron transfer process involving the biological oxygen transfer (Sechi, Rizzi *et al., *2009 ; Ramkumar *et al., *2008). Another exciting example of application for such polydentate ligand involves the synergic water activation, that occurs *via* so-called "remote metallic atoms". Such organometallic compounds are structurally deemed to promote or block the H—I activity (Zeng, Jiang *et al.*, 2008). These explanations clearly demonstrate that polydentate ligands are of specific interest in the field of bioorganometallic chemistry (Patil *et al.*, 2007; Pommier & Neamati, 2006).

We have undertaken the X-ray diffraction study of the title compound, in order to understand the molecular features which stabilize its conformation. The asymmetric unit contains two crystallographically independent molecules. Each molecule contains three aromatic rings (Fig. 1). The difference between the two molecules lies in the orientations of these three rings and carbonyl groups as shown in the fitting drawing (Fig. 2) obtained with PLATON (Spek, 2003). Thus in the first molecule (C11 to C146), the dihedral angles between the planes of benzene rings are: (C111–C116)/(C121–C126) = 75.41 (13)°, (C121–C126)/(C131–C136) = 75.94 (13)° and (C111–C116)/(C131–C136) = 22.36 (14)°. Whereas in the second molecule (C21 to C246), equivalent angles have as values 11.90 (15)°, 73.17 (17)° and 83.67 (15)°, respectively.

The planes of the two carbonyl groups (O11,O12,C141) and (O13,O14,C142) are twisted by a dihedral angles of 18.4 (4)° and 88.9 (4)°, respectively, with respect to the (C11,C14,N1) plane. In the second independent molecule, the dihedral angles between the two carbonyls (O21,O22,C241), (O23,O24,C242) and the (C21,C24,N2) plane are 25.9 (4)°, 87.1 (4)°, respectively. The angles between the planes of the two sites of disordered ethyl groups are 83.9 (15)° and 33.0 (2)° in the two molecules of the asymmetric unit.

In the crystal structure, weak intermolecular C—H···O hydrogen bonds (Table 1) link molecules into chains propagated along [10-1].

### Experimental

Synthesis of: 2-(4-trifloromethyl-benzylidene)-malonic acid diethyl ester]: To a solution of ethyl malonate (15 g, 93 mmol) in 40 ml of ethanol, were added the respective aldehyde (100 mmol), 1.5 ml of piperidine and 1 ml of glacial acetic acid. Then the mixture was stirred at refluxing temperature of ethanol for 12 h, until thin-layer chromatography indicated the complete consumption of the starting material. After removing solvent, the crude product was washed with a saturated solution of sodium bisulfite (20 ml). The product was extracted by diethyl ether (2x20 ml), dried with sodium sulphate and evaporated to give the respective pure oil.

To a solution of the 2-(4-trifloromethyl-benzylidene)-malonic acid diethyl ester (5 mmol) in water (25 ml) was added the dibenzylamine (6 mmol) in the presence of Acetic acid (0.1% mol) and the mixture and the stirring was continued at room temperature until the complete consume of the starting material (Pommier & Neamati, 2006).

After removing solvent, the crude products were dissolved in diethyl ether (2x40 ml) and washed with water until the pH became neutral. The organic solvent was dried with sodium sulphate and then evaporated to give the respective pure compounds as white solids. The residue was purified by recrystallization from a mixture ether/hexane (1:1) to give white solid in 96% yield.

Colourless crystals. 96% yield. Rf = 0.67 (ether/hexane: 1/1). M.p. = 102-104°C.

The structure of the title compound was deduced from ^1^H and ^13^C NMR, elemental analyses and fully confirmed by single-Crystal X-ray structure.

IR (KBr ) ν cm^-1^: 2841/2902 (CH); 1731(CO); 1585/1618 (C=C); 1257/1306(C—O); 1164.

RMN ^1^H (250 MHz, CDCl3) δ (ppm): 7,25-7,72 (m, 4H, aromat, Ph—CF_3_, ^3^J = 8,1 Hz);7.35 (m,10*H*, aromat, Ph ,^3^J = 6 Hz); 4.63 (d,1*H*, CF_3_PhC^3^H, ^3^J= 12 Hz); 4.4 (d, 1H, -C^2^H(CO_2_Et)2, ^3^J = 12 Hz); 3.0(d,1*H*, 2CH-Ph, ^3^J= 13,50 Hz ); 3.9 (d,1*H*, 2CHPh, ^3^J= 13.62 Hz ); 4.0 (dq, 2 H_AB_, OCH_2_CH_3_, J_AB_= 14,28 Hz, ^3^J = 7.15 Hz); 4.20/ 4.40 (dq, 2 H_AB_, OCH_2_CH_3_, J_AB_ = 14.28 Hz, ^3^J = 7.15 Hz); 1.28 (t, 3H, OCH_2_CH_3_ , ^3^J = 7.11 Hz); 1,0 (t, 3H, O CH_2_CH_3_, ^3^J = 7,11 Hz). RMN ^13^C (250 MHz, CDCl_3_) δ (ppm): 166.66/ 167.14 (2CO); 138.53 (C_quat_, C—CF_3_—Ph); 138,09 (C_quat_, Ph, para/ CF_3_); 128.17/125.01 ( _tert_, aromt); 130.22/129.43 (C_quat_, 2 C/ arm, 2Ph); 61.78/ 61.37 (C_sec_, 2CH_2_, ester); 61.28 (C_tert_, C^3^HPhCF_3_); 55,11 (C_tert_, C^2^H(CO_2_Et)_2_); 53.15/ 53.94 (C_sec_, 2CH_2_-Ph); 13.67 (C, OCH_2_CH_3_, ester); 13.91 (C, OCH_2_CH_3_, ester).

MS (IE) Calcd for [M]^+^ : 513.54, [M+H]^+^ = 514, [M—CH(CO_2_Et)_2_]^+^= 354 (100%).

Elemental analysis for C_29_H_30_F_3_NO_4_ Calcd (Found): C 67.82 (67.79), H 5.89 (5.87), N (2.73 (2.72).

The purity of the compound was checked by determining its melting point (104-106°C). Suitable single crystal of malonate derivative (I) was obtained by recrystallization from ethanol. A white-transparent crystal of C_29_H_30_F_3_NO_4_ having approximate dimensions of 0.43 × 0.25 × 0.17 mm was mounted on a glass fibre. All measurements were made in the φ and ω scans technique on a CCD X8 Bruker diffractometer with graphite monochromatized MoKα radiation at room temperature (296 (2) K).

### Refinement

All H atoms attached to C atoms were fixed geometrically and treated as riding with C—H = 0.96 Å (methyl), 0.97 Å (methylene) and 0.98 Å (methine) with *U*_iso_(H) = 1.2 or 1.5 *U*_eq_(C).

In asymmetric unit, two (of four) ethyl fragments were treated as disordered, and the occupancies of the major parts were initially refined, and then fixed to 0.53 and 0.64, respectively, in the final cycles of the refinement. Similarity restraints with tolerance s.u.s of 0.02 Å were applied to the chemically equivalent bond lengths and angles involving all disordered atoms. Refinement were carried out using the *DFIX*, SAME and PART instruction within *SHELXL97* (Sheldrick, 2008).

### Crystal data

**Table:**

C_29_H_30_F_3_NO_4_	*F*(000) = 2160
*M**_r_* = 513.54	*D*_x_ = 1.246 Mg m^−^^3^
Monoclinic, *P*2_1_/*c*	Melting point: 375 K
Hall symbol: -P 2ybc	Mo *K*α radiation, λ = 0.71073 Å
*a* = 13.4131 (3) Å	Cell parameters from 5382 reflections
*b* = 23.6608 (5) Å	θ = 2.5–25.4°
*c* = 17.3769 (3) Å	µ = 0.10 mm^−^^1^
β = 96.826 (1)°	*T* = 296 K
*V* = 5475.72 (19) Å^3^	Block, colourless
*Z* = 8	0.43 × 0.25 × 0.17 mm

### Data collection

**Table:**

Bruker APEXII CCD detector diffractometer	6912 reflections with *I* > 2σ(*I*)
Radiation source: fine-focus sealed tube	*R*_int_ = 0.041
graphite	θ_max_ = 26.0°, θ_min_ = 2.7°
ω and φ scans	*h* = −16→12
74220 measured reflections	*k* = −29→29
10790 independent reflections	*l* = −21→21

### Refinement

**Table:**

Refinement on *F*^2^	Primary atom site location: structure-invariant direct methods
Least-squares matrix: full	Secondary atom site location: difference Fourier map
*R*[*F*^2^ > 2σ(*F*^2^)] = 0.060	Hydrogen site location: inferred from neighbouring sites
*wR*(*F*^2^) = 0.184	H-atom parameters constrained
*S* = 1.02	*w* = 1/[σ^2^(*F*_o_^2^) + (0.0764*P*)^2^ + 3.875*P*] where *P* = (*F*_o_^2^ + 2*F*_c_^2^)/3
10790 reflections	(Δ/σ)_max_ = 0.011
709 parameters	Δρ_max_ = 0.51 e Å^−^^3^
10 restraints	Δρ_min_ = −0.41 e Å^−^^3^

### Special details

**Table:**

Experimental. The data collection nominally covered a sphere of reciprocal space, by a combination of five sets of exposures; each set had a different φ angle for the crystal and each exposure covered 0.5° in ω and 30 seconds in time. The crystal-to-detector distance was 37.5 mm.
Geometry. All esds (except the esd in the dihedral angle between two l.s. planes) are estimated using the full covariance matrix. The cell esds are taken into account individually in the estimation of esds in distances, angles and torsion angles; correlations between esds in cell parameters are only used when they are defined by crystal symmetry. An approximate (isotropic) treatment of cell esds is used for estimating esds involving l.s. planes.
Refinement. Refinement of *F*^2^ against ALL reflections. The weighted *R*-factor wR and goodness of fit *S* are based on *F*^2^, conventional *R*-factors *R* are based on *F*, with *F* set to zero for negative *F*^2^. The threshold expression of *F*^2^ > σ(*F*^2^) is used only for calculating *R*-factors(gt) etc. and is not relevant to the choice of reflections for refinement. *R*-factors based on *F*^2^ are statistically about twice as large as those based on *F*, and *R*- factors based on ALL data will be even larger.

### Fractional atomic coordinates and isotropic or equivalent isotropic displacement parameters (Å^2^)

**Table:**

	*x*	*y*	*z*	*U*_iso_*/*U*_eq_	Occ. (<1)
N1	0.21351 (13)	0.55799 (8)	0.47972 (11)	0.0377 (5)	
O12	0.13397 (17)	0.71894 (10)	0.37972 (14)	0.0757 (6)	
O13	0.20149 (13)	0.68514 (8)	0.56473 (11)	0.0531 (5)	
O14	0.08360 (14)	0.62505 (9)	0.59701 (11)	0.0610 (5)	
F11	0.0021 (2)	0.5779 (2)	0.07329 (13)	0.206 (2)	
F12	0.0673 (3)	0.49891 (18)	0.09034 (15)	0.1527 (13)	
F13	0.1539 (2)	0.56371 (14)	0.05959 (12)	0.1232 (10)	
C11	0.18374 (16)	0.60674 (11)	0.42886 (13)	0.0393 (5)	
H11	0.2426	0.6315	0.4314	0.047*	
C12	0.13542 (17)	0.51487 (11)	0.48334 (14)	0.0420 (6)	
H12A	0.1268	0.4938	0.4351	0.050*	
H12B	0.0722	0.5332	0.4896	0.050*	
C13	0.30752 (17)	0.53227 (11)	0.46200 (15)	0.0420 (6)	
H13A	0.3020	0.5241	0.4069	0.050*	
H13B	0.3170	0.4967	0.4895	0.050*	
C14	0.10159 (17)	0.64086 (11)	0.46267 (15)	0.0430 (6)	
H14	0.0388	0.6194	0.4535	0.052*	
C111	0.15593 (17)	0.59254 (11)	0.34376 (14)	0.0421 (6)	
C112	0.06123 (18)	0.57289 (12)	0.31431 (15)	0.0500 (7)	
H112	0.0125	0.5679	0.3476	0.060*	
C113	0.0385 (2)	0.56065 (14)	0.23677 (17)	0.0610 (8)	
H113	−0.0254	0.5479	0.2181	0.073*	
C114	0.1102 (2)	0.56730 (14)	0.18657 (16)	0.0592 (8)	
C115	0.2050 (2)	0.58634 (14)	0.21465 (16)	0.0585 (8)	
H115	0.2537	0.5907	0.1813	0.070*	
C116	0.22705 (19)	0.59887 (12)	0.29234 (15)	0.0491 (6)	
H116	0.2909	0.6118	0.3107	0.059*	
C117	0.0831 (3)	0.5552 (2)	0.1027 (2)	0.0897 (13)	
C121	0.16261 (17)	0.47463 (11)	0.54984 (14)	0.0434 (6)	
C122	0.15670 (18)	0.41678 (12)	0.53886 (16)	0.0490 (6)	
H122	0.1359	0.4024	0.4898	0.059*	
C123	0.18166 (19)	0.37990 (13)	0.60070 (19)	0.0573 (8)	
H123	0.1769	0.3411	0.5928	0.069*	
C124	0.2131 (2)	0.40070 (16)	0.67316 (19)	0.0644 (9)	
H124	0.2303	0.3761	0.7143	0.077*	
C125	0.2192 (2)	0.45806 (15)	0.68468 (17)	0.0618 (8)	
H125	0.2406	0.4722	0.7338	0.074*	
C126	0.1940 (2)	0.49465 (13)	0.62428 (16)	0.0530 (7)	
H126	0.1979	0.5334	0.6331	0.064*	
C131	0.39848 (16)	0.56895 (11)	0.48345 (14)	0.0387 (5)	
C132	0.47653 (18)	0.56848 (12)	0.43764 (16)	0.0495 (6)	
H132	0.4706	0.5472	0.3924	0.059*	
C133	0.56274 (19)	0.59933 (15)	0.45864 (18)	0.0619 (8)	
H133	0.6148	0.5982	0.4278	0.074*	
C134	0.5725 (2)	0.63169 (14)	0.52458 (19)	0.0622 (8)	
H134	0.6306	0.6526	0.5383	0.075*	
C135	0.4952 (2)	0.63286 (13)	0.57037 (17)	0.0573 (7)	
H135	0.5011	0.6547	0.6152	0.069*	
C136	0.40904 (18)	0.60163 (12)	0.54986 (15)	0.0476 (6)	
H136	0.3575	0.6026	0.5812	0.057*	
C141	0.0843 (2)	0.69809 (13)	0.42430 (18)	0.0564 (7)	
O11	0.00554 (18)	0.72241 (10)	0.45047 (17)	0.0879 (8)	
C143	−0.0206 (18)	0.7772 (5)	0.4092 (9)	0.099 (5)	0.47
H14A	0.0313	0.7881	0.3776	0.119*	0.47
H14B	−0.0840	0.7742	0.3762	0.119*	0.47
C144	−0.0277 (16)	0.8177 (5)	0.4702 (9)	0.217 (11)	0.47
H14C	−0.0817	0.8073	0.4990	0.326*	0.47
H14D	−0.0403	0.8546	0.4481	0.326*	0.47
H14E	0.0342	0.8182	0.5042	0.326*	0.47
C14B	−0.0790 (11)	0.7854 (5)	0.3714 (8)	0.174 (7)	0.53
H14F	−0.0454	0.7706	0.3300	0.261*	0.53
H14G	−0.0998	0.8236	0.3597	0.261*	0.53
H14H	−0.1368	0.7626	0.3773	0.261*	0.53
C14A	−0.0121 (16)	0.7845 (6)	0.4419 (9)	0.113 (6)	0.53
H14I	−0.0432	0.7999	0.4849	0.136*	0.53
H14J	0.0496	0.8048	0.4368	0.136*	0.53
C142	0.12649 (18)	0.64860 (12)	0.54960 (16)	0.0463 (6)	
C145	0.2325 (2)	0.69825 (15)	0.64581 (18)	0.0685 (9)	
H14K	0.3027	0.7090	0.6525	0.082*	
H14L	0.2249	0.6650	0.6772	0.082*	
C146	0.1703 (3)	0.7456 (2)	0.6722 (3)	0.1027 (14)	
H14M	0.1750	0.7779	0.6394	0.154*	
H14N	0.1945	0.7554	0.7248	0.154*	
H14O	0.1015	0.7338	0.6695	0.154*	
N2	0.38330 (14)	0.41631 (8)	0.17015 (11)	0.0379 (4)	
O22	0.66181 (15)	0.44204 (10)	0.08333 (13)	0.0670 (6)	
O23	0.54397 (17)	0.32714 (8)	0.10776 (12)	0.0624 (5)	
O24	0.54353 (15)	0.31656 (8)	0.23671 (12)	0.0604 (5)	
F21	0.4756 (3)	0.71223 (9)	0.11140 (17)	0.1370 (11)	
F22	0.56193 (16)	0.70396 (8)	0.22086 (16)	0.1008 (8)	
F23	0.40388 (17)	0.69796 (8)	0.21172 (16)	0.1031 (8)	
C21	0.47579 (17)	0.44015 (10)	0.14446 (13)	0.0353 (5)	
H21	0.4729	0.4318	0.0890	0.042*	
C22	0.29368 (18)	0.43168 (11)	0.11700 (14)	0.0431 (6)	
H22A	0.2941	0.4722	0.1082	0.052*	
H22B	0.2343	0.4228	0.1415	0.052*	
C23	0.36784 (19)	0.42964 (11)	0.25048 (14)	0.0438 (6)	
H23A	0.3486	0.4690	0.2539	0.053*	
H23B	0.4302	0.4241	0.2840	0.053*	
C24	0.56778 (17)	0.40876 (10)	0.18446 (14)	0.0396 (5)	
H24	0.5791	0.4205	0.2389	0.048*	
C211	0.48513 (17)	0.50390 (10)	0.15241 (13)	0.0358 (5)	
C212	0.51929 (18)	0.53005 (11)	0.22226 (14)	0.0423 (6)	
H212	0.5422	0.5081	0.2652	0.051*	
C213	0.5196 (2)	0.58816 (11)	0.22867 (16)	0.0485 (6)	
H213	0.5419	0.6051	0.2759	0.058*	
C214	0.4870 (2)	0.62105 (11)	0.16538 (16)	0.0482 (6)	
C215	0.4547 (2)	0.59608 (11)	0.09484 (16)	0.0496 (6)	
H215	0.4337	0.6183	0.0518	0.060*	
C216	0.45416 (18)	0.53773 (10)	0.08905 (14)	0.0413 (6)	
H216	0.4325	0.5209	0.0416	0.050*	
C217	0.4827 (3)	0.68374 (13)	0.1760 (2)	0.0680 (9)	
C221	0.28691 (18)	0.40196 (12)	0.04024 (14)	0.0457 (6)	
C222	0.2986 (3)	0.34475 (14)	0.03616 (19)	0.0717 (9)	
H222	0.3143	0.3239	0.0813	0.086*	
C223	0.2871 (3)	0.31745 (17)	−0.0360 (2)	0.0963 (13)	
H223	0.2951	0.2785	−0.0389	0.116*	
C224	0.2639 (3)	0.34860 (19)	−0.1022 (2)	0.0919 (12)	
H224	0.2568	0.3307	−0.1501	0.110*	
C225	0.2514 (3)	0.40534 (18)	−0.09839 (19)	0.0822 (11)	
H225	0.2348	0.4261	−0.1436	0.099*	
C226	0.2631 (2)	0.43214 (14)	−0.02777 (16)	0.0599 (8)	
H226	0.2549	0.4711	−0.0256	0.072*	
C231	0.28758 (19)	0.39275 (13)	0.27726 (14)	0.0494 (7)	
C232	0.2990 (2)	0.33483 (15)	0.2789 (2)	0.0697 (9)	
H232	0.3558	0.3183	0.2625	0.084*	
C233	0.2253 (3)	0.30143 (19)	0.3049 (3)	0.1002 (14)	
H233	0.2329	0.2624	0.3064	0.120*	
C234	0.1405 (3)	0.3259 (3)	0.3289 (2)	0.1038 (16)	
H234	0.0913	0.3032	0.3467	0.125*	
C235	0.1292 (3)	0.3825 (2)	0.3265 (2)	0.0914 (13)	
H235	0.0718	0.3989	0.3420	0.110*	
C236	0.2017 (2)	0.41593 (16)	0.30118 (17)	0.0656 (9)	
H236	0.1933	0.4549	0.3000	0.079*	
C242	0.55040 (19)	0.34538 (11)	0.18100 (16)	0.0470 (6)	
C241	0.66075 (19)	0.42246 (12)	0.14585 (17)	0.0492 (6)	
O21	0.74317 (16)	0.40944 (13)	0.19179 (15)	0.0946 (9)	
C243	0.8360 (4)	0.4181 (6)	0.1506 (4)	0.090 (3)	0.64
H24A	0.8342	0.3933	0.1061	0.109*	0.64
H24B	0.8398	0.4569	0.1332	0.109*	0.64
C244	0.9203 (4)	0.4050 (5)	0.2061 (4)	0.109 (3)	0.64
H24C	0.9181	0.4279	0.2515	0.163*	0.64
H24D	0.9812	0.4125	0.1841	0.163*	0.64
H24E	0.9180	0.3658	0.2201	0.163*	0.64
C24A	0.8544 (10)	0.4324 (5)	0.1860 (10)	0.128 (8)	0.36
H24F	0.8582	0.4645	0.1516	0.154*	0.36
H24G	0.8952	0.4379	0.2354	0.154*	0.36
C24B	0.8650 (19)	0.3778 (6)	0.1505 (12)	0.146 (8)	0.36
H24H	0.8623	0.3486	0.1887	0.219*	0.36
H24I	0.9283	0.3760	0.1300	0.219*	0.36
H24J	0.8115	0.3724	0.1094	0.219*	0.36
C245	0.5254 (3)	0.26672 (13)	0.0932 (2)	0.0752 (9)	
H24K	0.4916	0.2507	0.1346	0.090*	
H24L	0.4822	0.2618	0.0448	0.090*	
C246	0.6197 (3)	0.23760 (18)	0.0893 (3)	0.1093 (16)	
H24M	0.6521	0.2529	0.0475	0.164*	
H24N	0.6071	0.1980	0.0804	0.164*	
H24O	0.6623	0.2425	0.1372	0.164*	

### Atomic displacement parameters (Å^2^)

**Table:**

	*U*^11^	*U*^22^	*U*^33^	*U*^12^	*U*^13^	*U*^23^
N1	0.0311 (9)	0.0448 (12)	0.0382 (11)	−0.0060 (8)	0.0078 (8)	0.0023 (9)
O12	0.0774 (14)	0.0694 (15)	0.0820 (16)	0.0023 (12)	0.0158 (12)	0.0264 (13)
O13	0.0505 (10)	0.0579 (12)	0.0507 (11)	−0.0151 (9)	0.0055 (8)	−0.0077 (9)
O14	0.0595 (11)	0.0743 (14)	0.0534 (12)	−0.0140 (10)	0.0245 (9)	−0.0069 (10)
F11	0.151 (3)	0.404 (6)	0.0505 (14)	0.166 (3)	−0.0337 (15)	−0.042 (2)
F12	0.181 (3)	0.202 (4)	0.0710 (17)	−0.037 (3)	0.0001 (17)	−0.051 (2)
F13	0.1219 (19)	0.206 (3)	0.0443 (12)	0.0100 (19)	0.0196 (12)	−0.0045 (14)
C11	0.0329 (11)	0.0476 (15)	0.0381 (13)	−0.0077 (10)	0.0071 (9)	0.0021 (11)
C12	0.0366 (12)	0.0497 (15)	0.0395 (13)	−0.0102 (10)	0.0034 (10)	0.0022 (11)
C13	0.0387 (12)	0.0468 (15)	0.0412 (14)	−0.0007 (10)	0.0083 (10)	0.0001 (11)
C14	0.0328 (11)	0.0484 (15)	0.0481 (15)	−0.0059 (10)	0.0066 (10)	−0.0027 (12)
C111	0.0389 (12)	0.0480 (15)	0.0397 (14)	−0.0005 (10)	0.0057 (10)	0.0046 (11)
C112	0.0386 (13)	0.0710 (19)	0.0407 (14)	−0.0045 (12)	0.0063 (10)	−0.0025 (13)
C113	0.0451 (15)	0.088 (2)	0.0486 (17)	−0.0009 (14)	−0.0007 (12)	−0.0074 (15)
C114	0.0560 (16)	0.082 (2)	0.0394 (15)	0.0115 (15)	0.0029 (12)	−0.0009 (14)
C115	0.0568 (16)	0.077 (2)	0.0440 (16)	0.0044 (14)	0.0159 (13)	0.0072 (14)
C116	0.0407 (13)	0.0634 (18)	0.0443 (15)	−0.0045 (12)	0.0096 (11)	0.0045 (13)
C117	0.075 (2)	0.147 (4)	0.046 (2)	0.022 (2)	0.0023 (17)	−0.011 (2)
C121	0.0347 (12)	0.0536 (17)	0.0428 (14)	−0.0089 (11)	0.0078 (10)	0.0037 (12)
C122	0.0373 (12)	0.0577 (18)	0.0529 (16)	−0.0096 (11)	0.0095 (11)	0.0031 (13)
C123	0.0419 (14)	0.0547 (18)	0.077 (2)	−0.0031 (12)	0.0121 (13)	0.0169 (16)
C124	0.0469 (15)	0.087 (3)	0.060 (2)	−0.0028 (15)	0.0057 (13)	0.0303 (18)
C125	0.0586 (17)	0.083 (2)	0.0435 (16)	−0.0127 (15)	0.0039 (13)	0.0119 (16)
C126	0.0522 (15)	0.0607 (18)	0.0464 (16)	−0.0130 (13)	0.0068 (12)	0.0043 (13)
C131	0.0328 (11)	0.0470 (15)	0.0365 (13)	0.0011 (10)	0.0049 (9)	0.0065 (11)
C132	0.0392 (13)	0.0665 (19)	0.0438 (15)	0.0014 (12)	0.0094 (10)	0.0055 (13)
C133	0.0362 (14)	0.092 (2)	0.0584 (19)	−0.0052 (14)	0.0108 (12)	0.0165 (17)
C134	0.0393 (14)	0.079 (2)	0.066 (2)	−0.0162 (13)	−0.0054 (13)	0.0145 (17)
C135	0.0508 (15)	0.066 (2)	0.0523 (17)	−0.0068 (13)	−0.0070 (12)	−0.0023 (14)
C136	0.0383 (12)	0.0619 (18)	0.0425 (14)	−0.0027 (11)	0.0043 (10)	0.0007 (13)
C141	0.0518 (16)	0.0537 (18)	0.0621 (19)	−0.0016 (13)	0.0002 (13)	0.0017 (15)
O11	0.0780 (15)	0.0585 (15)	0.131 (2)	0.0232 (12)	0.0290 (15)	0.0073 (14)
C143	0.138 (11)	0.039 (5)	0.118 (15)	0.043 (6)	0.009 (11)	0.012 (7)
C144	0.37 (3)	0.088 (9)	0.210 (18)	0.103 (13)	0.118 (19)	−0.023 (10)
C14B	0.151 (10)	0.088 (8)	0.264 (18)	0.005 (7)	−0.058 (11)	0.077 (10)
C14A	0.123 (10)	0.126 (14)	0.094 (11)	0.051 (9)	0.029 (9)	0.015 (8)
C142	0.0394 (12)	0.0498 (16)	0.0512 (16)	−0.0022 (11)	0.0118 (11)	−0.0056 (13)
C145	0.0693 (19)	0.080 (2)	0.0548 (19)	−0.0153 (17)	−0.0003 (15)	−0.0133 (17)
C146	0.099 (3)	0.116 (4)	0.092 (3)	−0.004 (3)	0.008 (2)	−0.053 (3)
N2	0.0424 (10)	0.0403 (12)	0.0312 (10)	−0.0019 (8)	0.0055 (8)	0.0006 (9)
O22	0.0570 (12)	0.0791 (15)	0.0675 (14)	−0.0012 (10)	0.0181 (10)	0.0241 (12)
O23	0.0950 (15)	0.0400 (11)	0.0562 (12)	0.0020 (10)	0.0259 (11)	−0.0058 (9)
O24	0.0768 (13)	0.0461 (12)	0.0600 (13)	0.0070 (9)	0.0149 (10)	0.0181 (10)
F21	0.262 (4)	0.0399 (12)	0.111 (2)	0.0095 (16)	0.031 (2)	0.0165 (12)
F22	0.0904 (14)	0.0496 (12)	0.161 (2)	−0.0121 (10)	0.0093 (14)	−0.0315 (13)
F23	0.0920 (14)	0.0521 (12)	0.171 (2)	0.0132 (10)	0.0404 (15)	−0.0288 (13)
C21	0.0431 (12)	0.0317 (12)	0.0313 (12)	0.0008 (9)	0.0055 (9)	−0.0003 (10)
C22	0.0436 (13)	0.0460 (15)	0.0390 (14)	−0.0009 (11)	0.0018 (10)	−0.0003 (11)
C23	0.0475 (13)	0.0504 (16)	0.0342 (13)	−0.0032 (11)	0.0080 (10)	−0.0005 (11)
C24	0.0459 (13)	0.0351 (14)	0.0379 (13)	0.0039 (10)	0.0056 (10)	0.0015 (10)
C211	0.0381 (11)	0.0335 (13)	0.0368 (13)	0.0001 (9)	0.0079 (9)	−0.0010 (10)
C212	0.0508 (14)	0.0379 (14)	0.0377 (14)	−0.0028 (11)	0.0027 (10)	0.0006 (11)
C213	0.0602 (16)	0.0420 (16)	0.0436 (15)	−0.0040 (12)	0.0074 (12)	−0.0099 (12)
C214	0.0569 (15)	0.0334 (14)	0.0567 (17)	0.0008 (11)	0.0160 (12)	−0.0040 (12)
C215	0.0617 (16)	0.0395 (15)	0.0481 (16)	0.0054 (12)	0.0088 (12)	0.0077 (12)
C216	0.0528 (14)	0.0375 (14)	0.0341 (13)	0.0022 (11)	0.0073 (10)	0.0004 (10)
C217	0.082 (2)	0.0404 (17)	0.083 (2)	0.0040 (16)	0.0182 (18)	−0.0055 (17)
C221	0.0477 (14)	0.0494 (16)	0.0393 (14)	−0.0060 (11)	0.0025 (11)	−0.0033 (12)
C222	0.099 (2)	0.053 (2)	0.058 (2)	−0.0020 (17)	−0.0138 (17)	−0.0064 (15)
C223	0.136 (4)	0.057 (2)	0.087 (3)	0.009 (2)	−0.020 (2)	−0.024 (2)
C224	0.134 (3)	0.090 (3)	0.047 (2)	0.010 (2)	−0.007 (2)	−0.020 (2)
C225	0.116 (3)	0.087 (3)	0.0420 (18)	0.000 (2)	0.0004 (18)	0.0004 (18)
C226	0.0745 (19)	0.062 (2)	0.0422 (16)	−0.0043 (15)	0.0022 (13)	0.0014 (14)
C231	0.0486 (14)	0.066 (2)	0.0333 (13)	−0.0115 (13)	0.0039 (11)	0.0043 (12)
C232	0.0589 (18)	0.073 (2)	0.076 (2)	−0.0123 (15)	0.0031 (15)	0.0234 (18)
C233	0.095 (3)	0.088 (3)	0.116 (3)	−0.030 (2)	0.007 (3)	0.043 (3)
C234	0.081 (3)	0.149 (5)	0.083 (3)	−0.060 (3)	0.019 (2)	0.021 (3)
C235	0.069 (2)	0.145 (4)	0.064 (2)	−0.038 (2)	0.0262 (17)	−0.010 (2)
C236	0.0579 (17)	0.094 (2)	0.0477 (17)	−0.0152 (16)	0.0157 (13)	−0.0152 (16)
C242	0.0521 (14)	0.0379 (15)	0.0528 (17)	0.0074 (11)	0.0134 (12)	0.0034 (13)
C241	0.0470 (14)	0.0463 (16)	0.0546 (17)	0.0041 (11)	0.0077 (12)	0.0035 (13)
O21	0.0449 (12)	0.148 (3)	0.0906 (18)	0.0171 (13)	0.0057 (11)	0.0395 (17)
C243	0.040 (3)	0.169 (9)	0.062 (4)	0.010 (4)	0.007 (3)	0.016 (5)
C244	0.058 (4)	0.179 (8)	0.093 (5)	0.010 (5)	0.022 (3)	0.007 (6)
C24A	0.148 (16)	0.082 (10)	0.131 (14)	0.019 (9)	−0.083 (12)	0.017 (9)
C24B	0.21 (2)	0.087 (11)	0.155 (18)	0.026 (12)	0.063 (16)	−0.012 (11)
C245	0.098 (3)	0.0442 (18)	0.085 (2)	0.0035 (17)	0.0177 (19)	−0.0142 (17)
C246	0.085 (3)	0.070 (3)	0.171 (5)	0.023 (2)	0.007 (3)	−0.033 (3)

### Geometric parameters (Å, °)

**Table:**

N1—C13	1.465 (3)	N2—C23	1.470 (3)
N1—C12	1.469 (3)	N2—C22	1.472 (3)
N1—C11	1.479 (3)	N2—C21	1.479 (3)
O12—C141	1.187 (4)	O22—C241	1.183 (3)
O13—C142	1.329 (3)	O23—C242	1.337 (3)
O13—C145	1.454 (3)	O23—C245	1.468 (4)
O14—C142	1.197 (3)	O24—C242	1.197 (3)
F11—C117	1.265 (4)	F21—C217	1.302 (4)
F12—C117	1.361 (6)	F22—C217	1.331 (4)
F13—C117	1.295 (5)	F23—C217	1.332 (4)
C11—C111	1.519 (3)	C21—C211	1.518 (3)
C11—C14	1.538 (3)	C21—C24	1.534 (3)
C11—H11	0.9800	C21—H21	0.9800
C12—C121	1.508 (4)	C22—C221	1.501 (4)
C12—H12A	0.9700	C22—H22A	0.9700
C12—H12B	0.9700	C22—H22B	0.9700
C13—C131	1.508 (3)	C23—C231	1.502 (3)
C13—H13A	0.9700	C23—H23A	0.9700
C13—H13B	0.9700	C23—H23B	0.9700
C14—C141	1.515 (4)	C24—C242	1.518 (4)
C14—C142	1.519 (4)	C24—C241	1.520 (4)
C14—H14	0.9800	C24—H24	0.9800
C111—C116	1.391 (3)	C211—C216	1.384 (3)
C111—C112	1.392 (3)	C211—C212	1.391 (3)
C112—C113	1.377 (4)	C212—C213	1.379 (4)
C112—H112	0.9300	C212—H212	0.9300
C113—C114	1.382 (4)	C213—C214	1.376 (4)
C113—H113	0.9300	C213—H213	0.9300
C114—C115	1.382 (4)	C214—C215	1.383 (4)
C114—C117	1.487 (5)	C214—C217	1.497 (4)
C115—C116	1.380 (4)	C215—C216	1.384 (4)
C115—H115	0.9300	C215—H215	0.9300
C116—H116	0.9300	C216—H216	0.9300
C121—C122	1.383 (4)	C221—C222	1.366 (4)
C121—C126	1.395 (4)	C221—C226	1.385 (4)
C122—C123	1.394 (4)	C222—C223	1.402 (5)
C122—H122	0.9300	C222—H222	0.9300
C123—C124	1.371 (4)	C223—C224	1.371 (5)
C123—H123	0.9300	C223—H223	0.9300
C124—C125	1.373 (5)	C224—C225	1.355 (5)
C124—H124	0.9300	C224—H224	0.9300
C125—C126	1.372 (4)	C225—C226	1.374 (4)
C125—H125	0.9300	C225—H225	0.9300
C126—H126	0.9300	C226—H226	0.9300
C131—C136	1.382 (4)	C231—C232	1.379 (5)
C131—C132	1.389 (3)	C231—C236	1.383 (4)
C132—C133	1.379 (4)	C232—C233	1.383 (5)
C132—H132	0.9300	C232—H232	0.9300
C133—C134	1.371 (4)	C233—C234	1.384 (7)
C133—H133	0.9300	C233—H233	0.9300
C134—C135	1.380 (4)	C234—C235	1.349 (7)
C134—H134	0.9300	C234—H234	0.9300
C135—C136	1.382 (4)	C235—C236	1.366 (5)
C135—H135	0.9300	C235—H235	0.9300
C136—H136	0.9300	C236—H236	0.9300
C141—O11	1.330 (4)	C241—O21	1.321 (3)
O11—C14A	1.492 (12)	O21—C243	1.522 (6)
O11—C143	1.504 (10)	O21—C24A	1.602 (14)
C143—C144	1.440 (13)	C243—C244	1.430 (8)
C143—H14A	0.9700	C243—H24A	0.9700
C143—H14B	0.9700	C243—H24B	0.9700
C144—H14C	0.9600	C244—H24C	0.9600
C144—H14D	0.9600	C244—H24D	0.9600
C144—H14E	0.9600	C244—H24E	0.9600
C14B—C14A	1.430 (13)	C24A—C24B	1.447 (12)
C14B—H14F	0.9600	C24A—H24F	0.9700
C14B—H14G	0.9600	C24A—H24G	0.9700
C14B—H14H	0.9600	C24B—H24H	0.9600
C14A—H14I	0.9700	C24B—H24I	0.9600
C14A—H14J	0.9700	C24B—H24J	0.9600
C145—C146	1.501 (5)	C245—C246	1.449 (5)
C145—H14K	0.9700	C245—H24K	0.9700
C145—H14L	0.9700	C245—H24L	0.9700
C146—H14M	0.9600	C246—H24M	0.9600
C146—H14N	0.9600	C246—H24N	0.9600
C146—H14O	0.9600	C246—H24O	0.9600
			
C13—N1—C12	110.9 (2)	C145—C146—H14O	109.5
C13—N1—C11	112.11 (17)	H14M—C146—H14O	109.5
C12—N1—C11	115.09 (18)	H14N—C146—H14O	109.5
C142—O13—C145	116.9 (2)	C23—N2—C22	110.25 (19)
N1—C11—C111	115.3 (2)	C23—N2—C21	115.01 (18)
N1—C11—C14	109.64 (18)	C22—N2—C21	111.59 (18)
C111—C11—C14	112.57 (19)	C242—O23—C245	117.9 (2)
N1—C11—H11	106.2	N2—C21—C211	114.48 (18)
C111—C11—H11	106.2	N2—C21—C24	109.68 (18)
C14—C11—H11	106.2	C211—C21—C24	112.74 (19)
N1—C12—C121	111.26 (19)	N2—C21—H21	106.5
N1—C12—H12A	109.4	C211—C21—H21	106.5
C121—C12—H12A	109.4	C24—C21—H21	106.5
N1—C12—H12B	109.4	N2—C22—C221	113.6 (2)
C121—C12—H12B	109.4	N2—C22—H22A	108.8
H12A—C12—H12B	108.0	C221—C22—H22A	108.8
N1—C13—C131	113.6 (2)	N2—C22—H22B	108.8
N1—C13—H13A	108.8	C221—C22—H22B	108.8
C131—C13—H13A	108.8	H22A—C22—H22B	107.7
N1—C13—H13B	108.8	N2—C23—C231	110.9 (2)
C131—C13—H13B	108.8	N2—C23—H23A	109.5
H13A—C13—H13B	107.7	C231—C23—H23A	109.5
C141—C14—C142	109.5 (2)	N2—C23—H23B	109.5
C141—C14—C11	112.3 (2)	C231—C23—H23B	109.5
C142—C14—C11	111.1 (2)	H23A—C23—H23B	108.0
C141—C14—H14	107.9	C242—C24—C241	108.9 (2)
C142—C14—H14	107.9	C242—C24—C21	110.4 (2)
C11—C14—H14	107.9	C241—C24—C21	110.8 (2)
C116—C111—C112	117.7 (2)	C242—C24—H24	108.9
C116—C111—C11	119.6 (2)	C241—C24—H24	108.9
C112—C111—C11	122.7 (2)	C21—C24—H24	108.9
C113—C112—C111	121.1 (2)	C216—C211—C212	118.3 (2)
C113—C112—H112	119.5	C216—C211—C21	119.2 (2)
C111—C112—H112	119.5	C212—C211—C21	122.4 (2)
C112—C113—C114	120.3 (3)	C213—C212—C211	120.8 (2)
C112—C113—H113	119.9	C213—C212—H212	119.6
C114—C113—H113	119.9	C211—C212—H212	119.6
C115—C114—C113	119.6 (3)	C214—C213—C212	120.1 (2)
C115—C114—C117	121.1 (3)	C214—C213—H213	119.9
C113—C114—C117	119.2 (3)	C212—C213—H213	119.9
C116—C115—C114	119.8 (3)	C213—C214—C215	120.2 (2)
C116—C115—H115	120.1	C213—C214—C217	118.4 (3)
C114—C115—H115	120.1	C215—C214—C217	121.3 (3)
C115—C116—C111	121.5 (2)	C214—C215—C216	119.3 (2)
C115—C116—H116	119.3	C214—C215—H215	120.4
C111—C116—H116	119.3	C216—C215—H215	120.4
F11—C117—F13	110.7 (4)	C215—C216—C211	121.4 (2)
F11—C117—F12	104.0 (4)	C215—C216—H216	119.3
F13—C117—F12	99.9 (3)	C211—C216—H216	119.3
F11—C117—C114	114.4 (3)	F21—C217—F22	106.9 (3)
F13—C117—C114	115.2 (3)	F21—C217—F23	106.9 (3)
F12—C117—C114	111.1 (4)	F22—C217—F23	105.0 (3)
C122—C121—C126	118.0 (2)	F21—C217—C214	114.0 (3)
C122—C121—C12	121.0 (2)	F22—C217—C214	112.8 (3)
C126—C121—C12	121.0 (2)	F23—C217—C214	110.6 (3)
C121—C122—C123	120.6 (3)	C222—C221—C226	118.9 (3)
C121—C122—H122	119.7	C222—C221—C22	121.0 (3)
C123—C122—H122	119.7	C226—C221—C22	120.0 (3)
C124—C123—C122	120.2 (3)	C221—C222—C223	120.1 (3)
C124—C123—H123	119.9	C221—C222—H222	119.9
C122—C123—H123	119.9	C223—C222—H222	119.9
C123—C124—C125	119.7 (3)	C224—C223—C222	119.5 (4)
C123—C124—H124	120.2	C224—C223—H223	120.2
C125—C124—H124	120.2	C222—C223—H223	120.2
C126—C125—C124	120.5 (3)	C225—C224—C223	120.6 (3)
C126—C125—H125	119.8	C225—C224—H224	119.7
C124—C125—H125	119.8	C223—C224—H224	119.7
C125—C126—C121	121.0 (3)	C224—C225—C226	119.9 (3)
C125—C126—H126	119.5	C224—C225—H225	120.0
C121—C126—H126	119.5	C226—C225—H225	120.0
C136—C131—C132	118.3 (2)	C225—C226—C221	121.0 (3)
C136—C131—C13	121.9 (2)	C225—C226—H226	119.5
C132—C131—C13	119.8 (2)	C221—C226—H226	119.5
C133—C132—C131	120.6 (3)	C232—C231—C236	118.8 (3)
C133—C132—H132	119.7	C232—C231—C23	120.1 (3)
C131—C132—H132	119.7	C236—C231—C23	121.0 (3)
C134—C133—C132	120.7 (3)	C231—C232—C233	119.5 (4)
C134—C133—H133	119.7	C231—C232—H232	120.2
C132—C133—H133	119.7	C233—C232—H232	120.2
C133—C134—C135	119.3 (3)	C232—C233—C234	120.3 (4)
C133—C134—H134	120.3	C232—C233—H233	119.8
C135—C134—H134	120.3	C234—C233—H233	119.8
C134—C135—C136	120.2 (3)	C235—C234—C233	119.9 (3)
C134—C135—H135	119.9	C235—C234—H234	120.0
C136—C135—H135	119.9	C233—C234—H234	120.0
C135—C136—C131	120.9 (2)	C234—C235—C236	120.2 (4)
C135—C136—H136	119.5	C234—C235—H235	119.9
C131—C136—H136	119.5	C236—C235—H235	119.9
O12—C141—O11	125.0 (3)	C235—C236—C231	121.2 (4)
O12—C141—C14	126.2 (3)	C235—C236—H236	119.4
O11—C141—C14	108.8 (3)	C231—C236—H236	119.4
C141—O11—C14A	121.0 (9)	O24—C242—O23	125.7 (3)
C141—O11—C143	111.1 (8)	O24—C242—C24	123.9 (3)
C14A—O11—C143	22.8 (9)	O23—C242—C24	110.4 (2)
C144—C143—O11	104.8 (11)	O22—C241—O21	123.1 (3)
C144—C143—H14A	110.8	O22—C241—C24	126.1 (2)
O11—C143—H14A	110.8	O21—C241—C24	110.8 (2)
C144—C143—H14B	110.8	C241—O21—C243	110.9 (3)
O11—C143—H14B	110.8	C241—O21—C24A	127.0 (6)
H14A—C143—H14B	108.9	C243—O21—C24A	26.5 (5)
C14A—C14B—H14F	109.5	C244—C243—O21	106.2 (5)
C14A—C14B—H14G	109.5	C244—C243—H24A	110.5
H14F—C14B—H14G	109.5	O21—C243—H24A	110.5
C14A—C14B—H14H	109.5	C244—C243—H24B	110.5
H14F—C14B—H14H	109.5	O21—C243—H24B	110.5
H14G—C14B—H14H	109.5	H24A—C243—H24B	108.7
C14B—C14A—O11	100.2 (11)	C24B—C24A—O21	82.1 (12)
C14B—C14A—H14I	111.7	C24B—C24A—H24F	114.9
O11—C14A—H14I	111.7	O21—C24A—H24F	114.9
C14B—C14A—H14J	111.7	C24B—C24A—H24G	114.9
O11—C14A—H14J	111.7	O21—C24A—H24G	114.9
H14I—C14A—H14J	109.5	H24F—C24A—H24G	112.0
O14—C142—O13	125.5 (3)	C246—C245—O23	109.9 (3)
O14—C142—C14	124.2 (2)	C246—C245—H24K	109.7
O13—C142—C14	110.3 (2)	O23—C245—H24K	109.7
O13—C145—C146	110.5 (3)	C246—C245—H24L	109.7
O13—C145—H14K	109.5	O23—C245—H24L	109.7
C146—C145—H14K	109.5	H24K—C245—H24L	108.2
O13—C145—H14L	109.5	C245—C246—H24M	109.5
C146—C145—H14L	109.5	C245—C246—H24N	109.5
H14K—C145—H14L	108.1	H24M—C246—H24N	109.5
C145—C146—H14M	109.5	C245—C246—H24O	109.5
C145—C146—H14N	109.5	H24M—C246—H24O	109.5
H14M—C146—H14N	109.5	H24N—C246—H24O	109.5

### Hydrogen-bond geometry (Å, °)

**Table:**

*D*—H···*A*	*D*—H	H···*A*	*D*···*A*	*D*—H···*A*
C226—H226···O22^i^	0.93	2.60	3.323 (4)	135
C235—H235···O14^ii^	0.93	2.51	3.294 (4)	142

Symmetry codes: (i) −*x*+1, −*y*+1, −*z*; (ii) −*x*, −*y*+1, −*z*+1.
